# Lifespan trajectory of affect in Cornelia de Lange syndrome: towards a neurobiological hypothesis

**DOI:** 10.1186/s11689-019-9269-x

**Published:** 2019-06-07

**Authors:** Laura Groves, Joanna Moss, Hayley Crawford, Lisa Nelson, Chris Stinton, Gursharan Singla, Chris Oliver

**Affiliations:** 1 Cerebra Centre for Neurodevelopmental Disorders, School of Psychology, University of Birmingham, Edgbaston, B15 2TT UK; 2 Institute of Cognitive Neuroscience, University College London, 17 Queen Square, London, WC1N 3AR UK; 3 Faculty of Health and Life Sciences, Coventry University, Coventry, CV1 5FB UK; 4 Derby Royal Hospital, Uttoxeter Road, Derby, DE22 3NE UK; 5 Warwick Medical School, University of Warwick, Coventry, UK

**Keywords:** Mood, Affect, Trajectory, Cornelia de Lange syndrome, Fragile X syndrome

## Abstract

**Background:**

Depressive symptomology and low affect are comparatively common in individuals with genetic disorders such as Cornelia de Lange syndrome. However, lifespan trajectories and associated person characteristics have not been examined. In this study, the trajectories for affect and associated behavioural characteristics were investigated in individuals with Cornelia de Lange syndrome with individuals with fragile X syndrome (FXS) comparable for chronological age and total number of behavioural indicators of ASD included for the purpose of contrast.

**Methods:**

A 7-year longitudinal study of affect (mood, interest and pleasure) was conducted in individuals with CdLS (*n* = 44) and FXS (*n* = 95). The trajectories of low affect were explored, as well as associations between Time 1 behavioural characteristics and affect at Time 1 and Time 3 (7 years later).

**Results:**

The CdLS group were lower in mood than the FXS group overall (*p* < .001). Interest and pleasure scores showed a significant decline over the lifespan for individuals with CdLS (*p* < .001) but not the FXS group. Lower level of ability at Time 1 was associated with lower mood at Time 1 and Time 3 in the FXS group only. Higher levels of ASD symptomology at Time 1 were associated with low mood and interest and pleasure in both syndrome groups at Time 1 and Time 3. Greater insistence on sameness at Time 1 was associated with lower mood at Time 1 in the FXS group and lower interest and pleasure at Time 1 and Time 3 in the CdLS group.

**Conclusions:**

Low affect in specific genetic syndromes may be associated with differing lifespan trajectories and behavioural profiles. Specifically, individuals with CdLS appear at risk for experiencing declines in levels of interest and pleasure whereas individuals with FXS show no significant change in the level of affect with age.

## Background

Depression is comparatively prevalent in people with intellectual disability (ID) and genetic syndromes (estimated 4.0–22.3% [1, 2]) with long-term impact on the quality of life [3]. Clinical assessment of depression can be difficult due to limited expressive communication and compromised emotional recognition [4, 5]. Consequently assessments and research focus on observable signs indicative of low affect which encompasses behavioural indicators of mood, and interest and pleasure [6, 7]. For the purposes of this study, the term *affect* will be used as a global term for a pervasive emotional state under which *mood* and *interest and pleasure* constitute separate constructs. Mood and interest and pleasure may be considered as behavioural markers of depression and anhedonia described in clinical guidelines [7]. It is recommended that affect is monitored to optimise early intervention [6, 7], particularly in those at risk of decline. Lower adaptive ability, greater chronological age, and greater ASD symptomology specifically, have each been linked to low affect in people with ID [2, 5, 8–10]; although many of these associations warrant further investigation.

Research to date has predominantly assessed correlations between affect and person characteristics contemporaneously, with few studies evaluating characteristics that are predictive of future outcome levels of affect. This is particularly important as risk markers and developmental trajectories of affect in ID and genetic syndromes may differ from those for typically developing individuals with driving mechanisms remaining poorly understood. Specifically, it is unclear whether low affect emerges due to biological differences in genetic syndromes, whether risk markers such as lower adaptive ability, social impairments and social and economic adversity lead to vulnerability to environmental stressors and how environmental, biological and person characteristics interact.

Genetic syndromes offer a unique opportunity to explore the phenomenology, risk markers and trajectory of clinical outcomes such as low affect. These syndromes have specific and distinct genetic aetiologies with reports of variability in the prevalence and trajectories of affect even when matched for level of ID [11]. This suggests that differences in low affect may result, at least in part, from underlying biological mechanisms.

Cornelia de Lange syndrome (CdLS) is a cohesinopathy associated with ID with an estimated prevalence of 1:10,000–1:80,000 [12]. Abnormalities of the cohesin complex are associated with downregulation of proteins implicated in DNA maintenance and repair and increased global oxidative stress [13–15]. This increased sensitivity to damage and ageing at the cellular level may underpin observed behavioural and cognitive deterioration in individuals with CdLS [10, 11, 16–19]. Lower affect in older age in this syndrome has clear clinical significance and there may be a curvilinear trajectory, with 19–22 years being a critical period [10, 11]. However, no longitudinal study to date has been conducted to assess the conclusions drawn from the results of cross-sectional studies to [10]. In cross-sectional studies, the relationship with age remains significant, even after controlling for ASD symptomology. However, greater severity of ASD behaviours are also reported as associated with lower affect in these studies [10]. Indeed, at the behavioural level, ASD characteristics such as social withdrawal and isolation are often also reported as symptomatic of low affect, specifically social anhedonia [10, 11]. Given the high-reported prevalence of ASD symptomology in individuals with CdLS (approximately 43%; [20]) it is necessary to control for ASD so the presence of low affect can be assessed robustly.

Fragile X syndrome (FXS) has an estimated prevalence of 1:4000 males and 1:8000 females [21, 22], and is reported to be similar in the number of behavioural indicators of ASD to individuals with CdLS; hence, it affords a degree of control over co-varying ASD symptomatology [20, 23]. FXS results from the silencing of the *FMR1* gene leading to absence of the FMRP protein [24]. Overproduction of FMR1 mRNA in FMRP premutation carriers has been linked to emergence of fragile X-associated tremor/ataxia syndrome (FXTAS) which is a progressive disorder associated with cognitive decline intention tremor [25]. Although FXTAS has not been linked with full mutation FXS (with the expectation of a small number of mosaic individuals with an unmethylated full mutation (e.g. [26]) it is suggested that individuals with full mutation FXS may also be at risk for cognitive decline [25]. However, this and the developmental trajectories of other behaviours including low affect remain poorly understood.

This longitudinal study aimed to assess lifespan trajectories of affect in individuals with CdLS alongside individuals with FXS acting as a contrast syndrome with comparable levels of ASD symptomology. A secondary aim is to establish whether particular person characteristics are associated with either present or future levels of low affect in people with CdLS. Person characteristics were selected based on reported associations in previous literature and included chronological age, level of ability, ASD symptomology and insistence on sameness. We hypothesised that lower levels of affect would be associated with greater chronological age, lower levels of ability and greater number of ASD characteristics and insistence on sameness in CdLS. To achieve these aims, an informant-report questionnaire assessing behavioural indicators of low affect was employed due to difficulties assessing self-reported depression in these populations. Individuals with CdLS and FXS matched for chronological age and total number of behavioural indicators of ASD were assessed at baseline and followed up 3 years (Time 2; T2) and 7 years later (Time 3; T3).

## Methods

### Participants

As part of a larger study [10, 11, 23, 27, 28], caregivers of individuals with CdLS (*n* = 376) and FXS (*n* = 762) took part in a questionnaire survey. Caregivers were contacted through a database held at the Cerebra Centre for Neurodevelopmental Disorders at the University of Birmingham, the Cornelia de Lange Syndrome Foundation, UK and Ireland, or the Fragile X Society. Caregivers of 116 individuals with CdLS and 211 individuals with FXS consented to take part at T1. These individuals were invited to take part again at T2 and T3. Participants were included if they had a confirmed clinical diagnosis, reported no other chromosomal abnormalities, were aged over 4 years, had completed the Mood, Interest and Pleasure Questionnaire–Short [7, 29] at both T1 and T3, and had completed 75% of the other relevant questionnaires at T1. Missing data at T2 was permitted as T1–T3 scores represented the greatest measure of change over time and so allowed for the largest dataset possible. Furthermore, the statistical technique applied was robust against missing data [30] and T2 data were not included in the secondary analyses. Stem and leaf diagrams were inspected for extreme outliers with a small number of datum points identified. However, due to the extreme heterogeneity and intra-variability present within these syndrome groups, it was considered inappropriate to remove individuals from analyses as this may have comprised the generalisability of the sample. Forty-four individuals with CdLS and 95 individuals with FXS met criteria for inclusion (return rate from T1 data was 37.9% and 45.0%, respectively). Participant characteristics are shown in Table 1. Comparisons of the level of ability, ASD symptomology and level of affect at T1 showed no significant differences between individuals meeting inclusion criteria and those who did not (*p* > 0.05). The CdLS and FXS groups were comparable for chronological age and the total number of behavioural indicators of ASD as measured by the Social Communication Questionnaire [32] (*p* > 0.05). As expected, there were significantly more males in the FXS group. This was because, due to the phenotypic gender differences in FXS such that males are reported to have greater severity of behavioural and cognitive difficulties [33], only males were recruited. There were significant differences at all time points for self-help skills such that the FXS group was more able than the CdLS group; however, their scores remain suggestive of some degree of ID.

**Table 1:** Demographic information for participants with Cornelia de Lange and fragile X syndromes at T1, T2 and T3

		CdLS	FXS	*t*/*U*/*χ*^*2*^	*p* value
Time 1					
*n*		44	95		
Gender	% male	36.4	100.0	75.70	< 0.001
Age (years)	Mean *(SD)* range	18.39 *(10.04)* 4.02–40.75	17.29 *(9.14)* 6.61–47.49	1952.00	ns
T1 Self-help skills	% partly able/able	50.0	87.4	22.73	< 0.001
T1 SCQ^a^ total score	Mean *(SD)*	20.14 *(6.22)*	20.19 *(6.11)*	.086	ns
T2 Self-help skills	% partly able/able	45.9	87.7	29.86	< 0.001
T2 SCQ total score	Mean *(SD)*	20.26 *(6.08)*	20.12 *(6.64)*	.240	ns
T3 Self-help skills	% partly able/able	52.3	92.6	30.45	< 0.001
T3 SCQ total score	Mean *(SD)*	17.30 *(4.60)*	18.44 *(6.08)*	−.930	ns

*n* may vary due to missing data; ns = not significant

^a^The Autism Screening Questionnaire (ASQ [31]) was used at T1 and T2 as the SCQ was not available. The ASQ and SCQ differ on one item (item 20: social chat) and so for consistency this item was treated as missing data and was prorated for nonverbal participants (see [27])

### Measures

#### Demographic questionnaire

This questionnaire was used to collect background information including age, gender and diagnostic status.

#### Wessex Scale

The Wessex Scale [34] is an informant questionnaire used to assess self-help skills. It is reported to have good interrater reliability for both children and adults [34, 35].

#### Mood, Interest and Pleasure Questionnaire–Short form

The Mood, Interest and Pleasure Questionnaire–Short form (MIPQ-S [7, 29]) is an informant questionnaire assessing behavioural markers of low affect in individuals with ID across two subscales: mood, and interest and pleasure. Higher scores indicate the individual is higher in mood and taking more interest and pleasure from their environment. Internal consistency, test-retest reliability and interrater reliability are all reported to be good [7].

#### Social Communication Questionnaire

The Social Communication Questionnaire (SCQ [32]) originally called the Autism Screening Questionnaire (ASQ [31]) is an informant questionnaire which screens for the presence of behaviours associated with ASD across three subscales: communication, reciprocal social interaction, and repetitive and stereotyped behaviour. A higher score indicates a greater number of behaviours and responses that are consistent with a diagnosis of ASD. The sensitivity and specificity of the SCQ for screening for ASD and autism in populations with ID is good [32]. Internal consistency [32] and concurrent validity with the ADI and the ADOS [31, 36] are also reported to be good.

#### Repetitive Behaviour Questionnaire

The Repetitive Behaviour Questionnaire (RBQ, 26) is an informant questionnaire assessing the presence of repetitive behaviour in individuals with ID. There are five subscales: stereotyped behaviour, compulsive behaviour, restricted preferences, repetitive speech, and insistence on sameness. Higher scores indicate greater frequency of behaviours. The RBQ is reported to have good internal consistency, content validity, concurrent validity, test–retest reliability and inter-rater reliability [27]. Based on associations with affect reported in previous literature [10], only the insistence on sameness subscale was used.

### Procedure

At each time point, participants were sent a covering letter of invitation, an information sheet, a consent form and a questionnaire pack. Those who had not returned their pack within 4–6 weeks were sent a reminder in order to maximise response.

### Data analysis

The distribution of data was assessed using Kolmogrov-Smirnov tests and visual inspection of QQ plots. For non-normally distributed data, methods of transformation were unsuccessful in producing homogeneity of variance; therefore, non-parametric alternatives were employed. As multiple comparisons were being made, a conservative alpha (*p* < 0.01) was used throughout.

MIPQ-S scores for the CdLS and FXS group data were assessed for the two subscale levels: mood and, interest and pleasure. Mann-Whitney tests were used to assess group differences for level of mood, interest and pleasure at each time point and latent growth curve models with participants’ ages inputted as individually varying time points were fitted to the data in order to assess the development of these over time.

To identify how the broader behavioural profile may be associated with present and future levels of affect, Spearman’s Rho correlations were carried out between the MIPQ-S subscales and variables of interest for T1 and T3 scores. T3 data were selected over T2 data to give the longest timeframe of change and to limit the number of multiple comparisons. Variables were T1 data selected based on reported associations in previous literature [2, 5, 8–10]. Variables of interest included self-help scores as measured by the Wessex scale; total score and subscale scores (communication, reciprocal social interaction, repetitive behaviours) from the SCQ; insistence on sameness as measured by the RBQ.

## Results

### Comparison of affect between CdLS and FXS

Table 2 shows the medians and inter-quartile ranges of the MIPQ-S scores for total groups at T1, T2 and T3 for participants with CdLS and FXS. Comparisons of the syndrome groups showed that individuals with CdLS scored significantly lower on the mood subscale than the FXS group at all three time points. There were no significant group differences on the interest and pleasure subscale, although differences at T1 and T3 approached significance (*p* = .02 and .01, respectively) such that scores in the CdLS group were lower than the FXS group.

**Table 2:** Medians and inter-quartile ranges of MIPQ-S scores for the Cornelia de Lange and fragile X syndrome groups at T1, T2 and T3

	Median (inter-quartile range)		*U*	*p* value
	CdLS (*n* = 44)	FXS (*n* = 95)		
Time 1				
Mood	19.50 (16.73–21.00)	22.00 (20.00–23.00)	1144.50	< 0.001
Interest and pleasure	15.00 (12.00–18.00)	18.00 (14.00–20.00)	1571.00	ns
Time 2*				
Mood	18.00 (17.00–22.00)	21.00 (21.00–23.00)	987.00	0.003
Interest and pleasure	17.00 (12.00–19.00)	17.00 (14.00–19.00)	1325.00	ns
Time 3				
Mood	19.00 (17.00–21.00)	22.00 (20.00–23.00)	1064.50	< 0.001
Interest and pleasure	15.00 (11.00–18.00)	17.00 (13.82–19.00)	1537.50	ns

*n* with missing data: CdLS = 7, FXS = 14; ns = not significant

### Trajectories of affect in CdLS and FXS

**Fig. 1:**
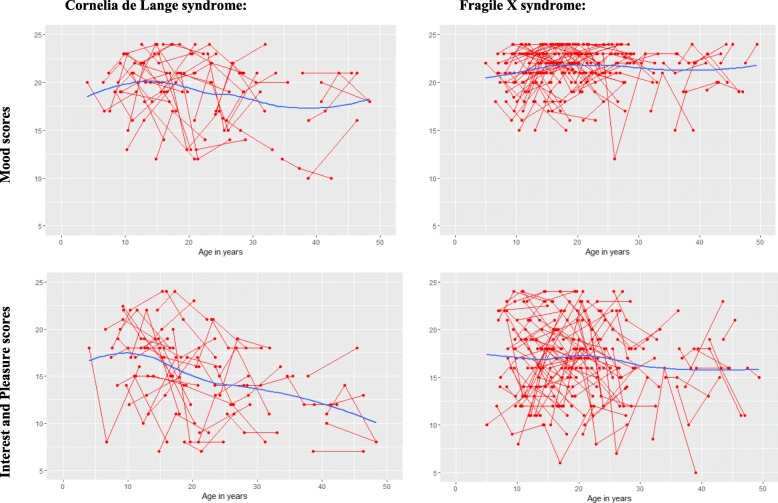
Individual and group trajectories of mood and interest and pleasure scores over time for the Cornelia de Lange and fragile X syndrome groups

To examine trajectories of affect in CdLS across the lifespan (see Fig. 1), latent growth curves were fitted to MIPQ-S data to assess the rate of change over time. The slopes for the FXS group’s mood and interest and pleasure scores were not significant (mood: est. = 0.01, SE = 0.02, ns; interest and pleasure: est. = − 0.04, SE = 0.03, ns). The slopes for the CdLS group’s mood scores were also not significant (est. = − 0.06, SE = 0.04, ns), However, the CdLS group’s interest and pleasure scores did significantly decrease by approximately 0.16 per year (est. = − 0.16, SE = 0.03, *p* < 0.001) suggesting a significant deterioration in the level of interest and pleasure individuals experienced over time. There was no significant variability for the slope (est. = 0.26, SE = 0.16, ns) indicating that all participants’ scores changed over time at the same rate.

### Associations between behavioural characteristics and affect in CdLS and FXS

To identify which behavioural characteristics may be associated with present and future levels of low mood, correlation analyses were conducted between T1 participant characteristics and T1 and T3 MIPQ-S scores (see Table 3). For the CdLS group, greater insistence on sameness, greater difficulties with social interaction and total ASD characteristics at T1 were significantly correlated with lower interest and pleasure scores at T1. Only T1 ASD total scores and insistence on sameness were significantly correlated with interest and pleasure outcome at T3. Lower mood scores at T1 were significantly correlated with higher levels of repetitive behaviour and total ASD characteristics at T1; no significant correlations were found between any T1 participant characteristics and mood at T3. For T1 FXS data, lower interest and pleasure scores were significantly correlated with greater insistence on sameness, greater difficulties with social interaction and total ASD characteristics. Only T1 ASD total score and the social interaction subscale were correlated with interest and pleasure outcome at T3. Low mood at T1 was correlated with lower self-help skills, greater difficulties with social interaction and total ASD characteristics at T1; however, only T1 self-help skills correlated with outcome at T3.

**Table 3:** Correlations of mood and interest and pleasure subscales at Time 1 and Time 3 with variables of interest at Time 1 for the Cornelia de Lange and fragile X syndrome groups

	Self-help skills	Autism spectrum disorder				Insistence on sameness
		Comm.	Social interaction	Repetitive behaviour	Total score	
CdLS						
Time 1						
Mood	.20	− .19	− .29	− .51**	− .42*	− .18
Interest and pleasure	− .01	− .34	− .54*	− .16	− .59**	− .41*
Time 3						
Mood	− .12	− .16	− .16	− .26	− .19	− .34
Interest and pleasure	− .11	− .17	− .39	− .24	− .48*	− .42*
FXS						
Time 1						
Mood	.38*	− .21	− .41**	− .27	− .36*	− .38*
Interest and pleasure	.12	− .20	− .31*	− .16	− .28*	.05
Time 3						
Mood	.28*	− .21	− .25	−.09	−.22	−.11
Interest and pleasure	.08	− .23	− .42**	−.07	−.33*	−.05

*p < 0.01; **p < 0.001

## Discussion

In this study, lifespan trajectories of affect in groups of individuals with CdLS and FXS were examined utilising longitudinal methodologies. In addition, associations between affect and self-help skills, ASD symptomology and insistence on sameness were assessed. This study is the first to evaluate whether these variables are associated with outcome for affect 7 years later.

### Prevalence and trajectories of affect in CdLS and FXS

Syndrome group comparisons showed that the CdLS group had significantly lower mood scores than the FXS group, and neither group showed significant change over time. Conversely, interest and pleasure scores did not significantly differ when participants were assessed at a group level. The CdLS group showed a significant decline in this score over the lifespan, this was not the case for participants with FXS. This pattern of divergent trajectories of mood and interest and pleasure in CdLS has not been described in previous research, which may be because these constructs are often assessed together [9, 10, 18], thus masking potential differences that occur at a more refined level. Our findings indicate that the reported decline in affect is primarily driven by decreasing levels of interest and pleasure specifically, rather than affect as a whole. The current study findings are consistent with previous research reports of increased prevalence of low affect in individuals with CdLS [16, 18].

Research in CdLS has identified approximately 15–22 years as being a key period for the onset of emotional change [9, 10, 18] but this was not statistically significant in the slope of the growth curves in the current study. However, visual inspection of the interest and pleasure graph for the CdLS group (Fig. 1) does show that scores remained relatively stable until the ages of approximately 12–15 years where a downward trajectory of scores appears. This effect may not have been identified as significant due to the reduced statistical power from the small *n* value and the inclusion of three time-points meaning only a linear model could be fitted to the data. In addition, there was notable intra-individual variability in scores which may have somewhat attenuated the gradient of the growth curve slope. It was not possible to determine how significant this variability was; however, this pattern supports previous descriptions of fluctuating mood in CdLS [10, 37]. It is unclear whether these represent discrete clinically significant episodes of low affect, or periods of time where low affect is particularly salient to caregivers beyond a persistent baseline low affect. Future research is required to understand the significance of these more transient periods and risk factors that may be implicated, as well as the clinical significance of the persistent low affect reported here.

### Behavioural correlates of affect

To understand the mechanisms that may underlie the different presentations of affect in these syndrome groups, and the differential trajectories of mood and declining interest and pleasure scores in the CdLS group, it is useful to examine associations with participant characteristics. To do this, correlations were conducted between T1 and T3 measures of affect and behavioural variables at T1. These showed no association between self-help skills and either subscale from the MIPQ-S for the CdLS group and for the interest and pleasure scores in the FXS group. Self-help scores were associated with mood in individuals with FXS at T1 and T3 such that individuals with lower ability were reported to have lower mood. This indicates that level of ability may be a marker for identifying individuals with FXS at risk of experiencing low mood. However, as no comparison group with similar level of ability to the FXS group was included in this study, future research is required to evaluate the utility of using indicators of level of ability for this purpose.

For the CdLS group, greater insistence on sameness and a number of ASD characteristics were associated with lower interest and pleasure scores at T1 and outcome scores at T3, which is consistent with reports from previous cross-sectional studies [9, 10, 37]. Our findings expand upon this evidence base by demonstrating that these behaviours are also associated with future interest and pleasure scores, 7 years later. No significant correlations with insistence on sameness were found within the FXS group. However, ASD total scores were significantly associated with interest and pleasure scores at T1 and outcome scores at T3. This suggests that ASD symptomology is strongly related to interest and pleasure in these genetic syndromes but associations with insistence on sameness may be specific to individuals with CdLS.

Caution should be exercised when interpreting associations between interest and pleasure and measures of ASD symptomology given the similarities in behavioural manifestations of these, such as social withdrawal and isolation [7]. This is particularly significant given that the social interaction skills were associated with mood, and interest and pleasure scores of participants with CdLS (interest and pleasure at T1) and FXS (both subscales at T1 and interest and pleasure at T3) and so may suggest levels of affect were confounded by presence of ASD characteristics. However, despite the CdLS and FXS groups being comparable for the total number of behavioural indicators of ASD at each time point, significant group differences for mood were found, as well as a decline in interest and pleasure scores over time in the CdLS group only. This suggests that ASD symptomology cannot completely account for levels of affect in the two syndrome groups. In order to understand the presentation of interest and pleasure in CdLS it is important to consider the roles of age and insistence on sameness in this group. Insistence on sameness is thought to be associated with low interest and pleasure because when changes in routines or the environment are made in conflict with an individual’s preference for sameness, this can be emotionally distressing and may lead to an anxiety response and withdrawal from situations where change may occur [9, 37]. Thus, both individuals with CdLS and FXS have increased vulnerability to environmental stressors due to presence of ASD symptomology. However, the influence of additional factors, such as insistence on sameness and chronological age in CdLS may make this more difficult for this group and could represent a syndrome-specific difficulty.

A further consideration is due to degree of intellectual disability varying so substantially in CdLS [15], it is important to consider that individuals with greater cognitive ability may present with differing trajectories to those with lower cognitive skills. The cognitive reserve hypothesis postulates that individual differences in cognitive processing may afford individuals with greater cognitive ability, an increased capacity to manage or compensate for the clinical manifestations of neurodegenerative disorders despite presenting with associated neuropathology [38, 39]. This is important to consider in CdLS and may in part explain the sudden onset of difficulties in certain individuals, and any individual variability of onset of difficulties.

### Strengths and limitations

A particular strength of this study is the 7-year follow-up period as no other study to date has examined changes in affect in individuals with CdLS and FXS over such a long timeframe. The inclusion of both groups meant that level of affect could be assessed independently of ASD characteristics, which has been described as having a similar behavioural presentation to low affect [7, 10]. However, the inclusion of the FXS group may have limited the interpretation of findings. Whilst cross syndrome contrasts are advantageous for a number of reasons [40] and can indicate direction of difficulties, ascertaining within this which group would be considered ‘atypical’ without reference to an additional normative group is challenging. However, previous literature does point towards low affect in CdLS and relatively ‘typical’ levels of affect in FXS compared to other syndrome groups [10, 11]. Nonetheless, it is important to consider this limitation when interpreting findings as the FXS group were more able than the CdLS group which, given the associations between level of ability and low mood in individuals with idiopathic ASD [2], could be a confounding variable. However, as self-help skill scores were not associated with the variability of mood or interest and pleasure scores in the CdLS group this is unlikely to have impacted the findings of this study. Future studies should aim to include groups comparable to the CdLS group on measures of ability in order to elucidate the impact of this on affect.

The application of the latent growth curve analysis strengthens the findings of this study as it allowed trajectories of affect to be assessed with the consideration of the participant’s exact age at each time point and so was sensitive to atypical and variable trajectories. However, as chronological age was inputted into the growth curves as individually varying time points, the results may have been vulnerable to cohort effects as this method combines longitudinal and cross-sectional analyses of the data. A further limitation is that the predictor variables explored are not exhaustive and there are likely to be other variables associated with affect, or which mediate their interactions with other behavioural characteristics. Most notably, medication use may have impacted the presentation of affect and management of this over time. Finally, the inclusion of informant measures only is a limitation and further studies should aim to incorporate observational assessments of affect.

### Potential mechanisms

There is an emergent literature indicating change over time in individuals with CdLS. Changes manifest as a decline in cognitive and executive function (EF) skills and increasing ASD symptomology and anxiety [10, 18, 19, 41], which when considered alongside the additional decreases in interest and pleasure shown in this study, may be evident of a broader developmental decline. In order to understand how these changes are linked, a hypothesised model is proposed.

In CdLS, there are reports of premature ageing in both the physical phenotype and at the cellular level which could provide preliminary evidence of neurodegeneration [7, 13, 14]. Most notably, CdLS cell lines are reported to show increase in global oxidative stress which is implicated in other neurodegenerative disorders such as Alzheimer’s disease [42]. Neurodegenerative disorders, such as Parkinson’s disorder, are associated with functional and structural decline in the prefrontal cortex, such as cortical thinning [43]. The prefrontal cortex is implicated in EF, and there are reports of declining EF skills in individuals with neurodegenerative disorders [43]. Few studies examining brain function have been reported in CdLS, of those which have frontal lobe hypoplasia is described [44]. We propose that structural and functional prefrontal impairments underlie difficulties in EF in CdLS. Because of this, situations which present high-cognitive load for example those which are unfamiliar or unpredictable may be difficult for individuals to manage. Preference for sameness may offer the individual a sense of predictability and consistency which lessens the cognitive load required to navigate daily life. Thus, insistence on sameness may be heightened in individuals where EF difficulties are greater. We further propose that executive dysfunction underpins the behavioural change in this group, mediated by insistence on sameness. This fits with the associations between chronological age and insistence and sameness and interest and pleasure reported here, and also associations between insistence and sameness and anxiety in the general literature [45–47]. Further work is required to corroborate this hypothesis, as well as to determine whether it may be impaired development or degeneration driving observed change in CdLS.

## Conclusions

In summary, this study has shown that individuals with CdLS are at risk for a decline in interest and pleasure. This should be considered by professionals working with individuals by monitoring levels of interest and pleasure over time with an aim to provide early intervention and support for those at risk of experiencing a decline. Specifically, these are individuals with a greater number of behavioural indicators of ASD and greater insistence on sameness. Conversely, the FXS group showed no sign of decline and overall showed higher levels of mood than the CdLS group. It is of note that the oldest individuals included in this study were aged 54 years which may be too young to detect change in the FXS group as mean age at onset of neurodegeneration disorders in the general population is approximately 60–70 years and 50–60 years for onset of FXTAS in fragile X premutation carriers [25, 48–50]. However, this makes the observed early decline of the CdLS group particularly significant and thus increases the importance of professionals monitoring behaviour and participant characteristics associated with low affect in CdLS from an early age. A hypothesised role of the prefrontal cortex in these changes has been proposed; however, future studies are required to corroborate and evaluate this. To conclude, this study highlights the importance of examining and understanding trajectories of affect in individuals with ID of different aetiology as clear differences in the presentation of this across syndromes has been identified.

## Data Availability

The data that support the findings of this study are not available due to them containing information that could compromise research participant consent.
